# Innovative and disruptive technologies to prescribe, encourage, and evaluate physical exercise in healthy adults: a protocol of exploratory study followed by a noninferiority, investigator-blinded randomized clinical trial

**DOI:** 10.1186/s13063-023-07747-6

**Published:** 2023-10-30

**Authors:** Fernanda Laís Loro, Riane Martins, Cintia Laura Pereira de Araújo, Lucio Rene Prade, Denis Lima do Rosário, Marcos César da Rocha Seruffo, Italo Adriano Moraes de Freitas, Jéferson Nobre, Cristiano Bonato Both, Pedro Dal Lago

**Affiliations:** 1 Universidade Federal de Ciências da Saúde de Porto Alegre, Sarmento Leite Street, 245, Porto Alegre, RS CEP: 90050-170 Brazil; 2 Universidade do Vale do Rio dos Sinos, Unisinos Avenue, 950, São Leopoldo, RS CEP: 93022-750 Brazil; 3 Universidade Federal do Pará, Augusto Correa Street, 01, Belém, PA CEP 66075110 Brazil; 4 Ludus Studio, Quartorze Street, 6, Coqueiro Ananindeua, PA CEP 67113-510 Brazil; 5 Universidade Federal do Rio Grande do Sul, Bento Gonçalves Avenue, 95000, Porto Alegre, RS CEP 91501970 Brazil

**Keywords:** Exercise training, Exercise intensity, Mobile applications, Sedentary lifestyle

## Abstract

**Background:**

Cardiovascular diseases are a leading cause of mortality worldwide. A significant contributing factor to this mortality is the lack of engagement in preventive activities. Consequently, strategies for enhancing adherence to and duration of physical activity (PA) have become pivotal. This project aims to create and validate innovative, disruptive, and secure technologies that ensure appropriate exercise intensity, bolster adherence to PA, and monitor health biomarker responses pre-, during, and post-physical activity.

**Methods:**

This exploratory study, followed by a noninferiority, investigator-blinded randomized clinical trial, will be divided into three phases: (1) development and validation of a sensor for real-time biofeedback during a functional assessment test; (2) integration of biofeedback and gamification into an app for the structured prescription of physical training within a controlled setting; and (3) implementation of biofeedback and gamification into an app for the prescription and monitoring of physical training in an uncontrolled setting. Phase 1 entails a validation test of a biosensor—monitoring heart rate (HR) and steps—during a modified shuttle walk test. In phase 2, the biosensor interfaces with a gamified smartphone application. The training regimen spans 6 weeks, 5 days weekly, with each session lasting 60 min: a five-min warm-up involving stationary gait, followed by 50 min of training at the target HR on the step and concluding with a five-min cool-down at a stationary pace. After 6 weeks of training, a new functional capacity test is conducted. Phase 3 involves an investigator-blinded, randomized clinical trial to demonstrate noninferiority. Participants are randomly assigned to either the intervention group (IG) or the control group (CG). IG participants practice exercise using the gamified application in an uncontrolled environment according to the prescribed method outlined in phase 2. CG participants receive PA practice guidelines exclusively.

**Discussion:**

Anticipated outcomes include improved exercise adherence through the gamified application, better maintenance of prescribed exercise intensity, and enhanced health biomarkers. The results of this study will inform health-related decision-making.

**Trial registration:**

The study protocol received approval from the Ethics Committee of Universidade Federal de Ciências da Saúde de Porto Alegre (54,492,221.80000.5345) and has been registered with the Brazilian Registry of Clinical Trials (ReBEC, RBR-359p69v).

## Administrative information

Note: The numbers in curly brackets in this protocol refer to SPIRIT checklist item numbers. The order of the items has been modified to group similar items (see)

**Table:** 

Title {1}	Innovative and disruptive technologies to prescribe, encourage and evaluate physical exercise in healthy adults: A protocol of exploratory study followed by a noninferiority, investigator-blinded randomized clinical trial.
Trial registration {2a and 2b}.	The Brazilian Registry of Clinical Trials (ReBEC). Identifier: RBR-359p69v, registered on August, 21, 2023.
Protocol version {3}	Version 1.0, registered August, 21, 2023.
Funding {4}	This work is supported by the Fundação de Amparo à Pesquisa do Estado de São Paulo (FAPESP) grant 2020/05155–6.
Author details {5a}	Fernanda Laís Loro^1^, Riane Martins^1^, Cintia Laura Pereira de Araújo^1^, Lucio Rene Prade^2^, Denis Lima do Rosário^3^, Marcos Cesar da Rocha Serruffo^3^, Italo Adriano Moraes de Freitas, Jéferson Nobre^4^, Cristiano Bonato Both^2^, Pedro Dal Lago^1^ 1- Universidade Federal de Ciências da Saúde de Porto Alegre (UFCSPA). Porto Alegre—Brazil 2- Universidade do Vale do Rio do Sinos (UNISINOS). Porto Alegre—Brazil 3- Universidade Federal do Pará (UFPA). Belém—Brazil 4- Universidade Federal do Rio Grande do Sul (UFRGS). Porto Alegre—Brazil
Name and contact information for the trial sponsor {5b}	São Paulo Research Foundation (FAPESP). Pio XI Streat, 1500—Alto da Lapa – CEP 0546–901- São Paulo -SP—Brazil. Telephone: + 55 011 3838 4000. Email address: converse2@fapesp.br.
Role of sponsor {5c}	The sponsor has no role in the research.

## Introduction

### Background and rationale {6a}

Roughly 9% of premature deaths worldwide, equivalent to 5.3 million individuals, are directly attributed to physical inactivity [1]. Recently, the World Health Organization (WHO) estimates highlight that over 1.4 billion adults face the risk of developing or worsening conditions linked to inactivity [2]. However, since 2001, global levels of physical activity (PA) have stagnated. In 2016, more than a quarter (1.4 billion) of the global adult population was physically inactive, falling short of WHO’s aerobic exercise recommendations [3]. This sedentary lifestyle substantially heightens the risk of noncommunicable diseases and premature death [2]. The scientific community is intently focused on sedentary behaviour due to its detrimental link to health status, the rise of chronic noncommunicable diseases, and its prevalence across populations. Solid epidemiological evidence underscores the connection between sedentary behaviour duration and all-cause mortality rates [4].

According to WHO, up to 5 million deaths worldwide could be averted annually if people were more active and less sedentary. Moreover, engaging in physical exercises is pivotal for averting the onset and managing chronic noncommunicable diseases such as ischemic heart disease, stroke, chronic obstructive pulmonary disease, type 2 diabetes, and certain cancer types. In response, the WHO introduced a global action plan in 2018 aimed at reducing physical inactivity by 15% by 2030 [5]. Consequently, developing strategies to stimulate practice, participation, and adherence to physical activities within sedentary populations is imperative. The potential of gamified interventions is promising in promoting physical activity across diverse demographics [6]. These interventions leverage gaming techniques to infuse activities with playfulness, challenges, relevant rewards, and increased participant motivation, fostering lasting habits [7–9].

In this context, games offer a means to engage individuals in physical activity and educate them on cultivating regular exercise habits [10, 11]. For instance, games addressing healthy eating and exercise have been developed to combat childhood obesity [10]. Additionally, studies highlight the value of gaming in aiding respiratory rehabilitation [11].

Embracing new technologies, wearable sensors for tracking biological signals, and wireless sensor networks like low-power wide area network (LPWA) show promise in promoting physical exercise [12]. Their affordability, sustainability, and integration with the Internet of Things (IoT) make them accessible, especially when integrated into smartphones. Furthermore, they facilitate noninvasive biofeedback of physiological variables such as heart rate (HR) [13].

There are several brands of wearable devices that measure HR; however, they are expensive for the population [14, 15]. In addition to this technology, gamification has been the subject of attention and study as a way of encouraging physical exercise since 2010. A gap in the literature refers to the integration of gamification with wearable sensors that promote HR biofeedback [16].

### Objectives {7}

The primary goals of this study encompass three key objectives: (1) innovative technology development: the first objective revolves around the creation and validation of innovative and secure technologies—a biosensor and an app; these technologies are engineered to guarantee optimal intensity during prescribed physical exercises, setting the stage for a safe and effective workout experience; (2) enhanced adherence and monitoring: the second aim focuses on fostering heightened adherence to physical exercise routines while simultaneously monitoring health biomarker responses; these responses, encompassing heart rate and step data, will be tracked before, during, and after each session, providing valuable insights into the impact of the exercise regimen; and (3) sedentary lifestyle reduction: the third objective seeks to curb the prevalence of sedentary lifestyles within the population. By promoting engagement in physical activity through our technologies, we aim to contribute to a meaningful reduction in sedentary behaviours and their associated health risks.

### Trial design {8}

This study employs an exploratory design, succeeded by a noninferiority, investigator-blinded randomized clinical trial, to assess the viability of a gamified application prototype. The prototype incorporates a biofeedback system to guide physical exercises, ensuring adherence to prescribed intensity levels. The study protocol adheres to the guidelines set by Standard Protocol Items: Recommendations for Interventional Trial (SPIRIT) [17] and was registered in the Brazilian Registry Clinical Trials (ReBEC) by the identifier RBR-359p69v.

**Fig. 1:**
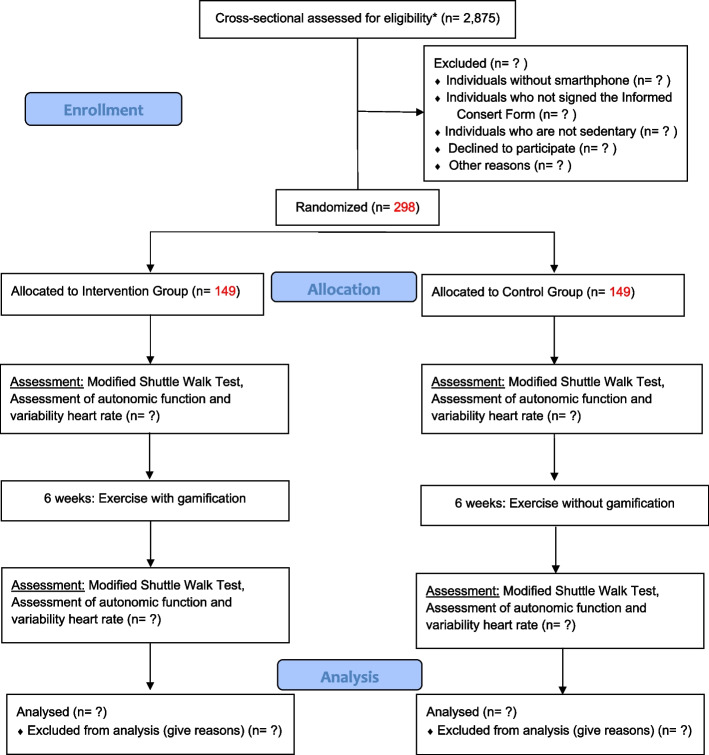
Flow Chart of participants. Asterisk (*) symbol indicates the following: from the 2875 individuals interviewed using the IPAQ, those identified as sedentary will undergo our eligibility screening. Subsequently, the qualified participants will be randomized

The study unfolds in three distinct phases: (1) validation of biosensor and app: this phase involves validating the functionality of the biosensor and app within the context of a functional assessment test; (2) validation of biofeedback and gamified application: the second phase encompasses the validation of the biofeedback and gamified application for prescribing and conducting physical training in a controlled environment; (3) validation of gamified application in noncontrolled setting: the final phase entails validating the gamified application for prescription and physical training within a noncontrolled environment. This validation will be executed through a noninferiority, investigator-blinded randomized clinical trial. The flowchart depicting the progression of trial participants can be found in Fig. 1.

This study received approval from the Research Ethics Committee of UFCSPA prior to data collection, with registration number 54492221.80000.5345. The research team assumes responsibility for ensuring the integrity of this study.

## Methods: participants, interventions, and outcomes

### Study setting {9}

The sample will be conveniently recruited through a social media invitation in a city located in southern Brazil. This protocol was designed exclusively by researchers, without input from community members or patients.

### Eligibility criteria {10}

The recruitment of individuals will adhere to specific inclusion criteria: (i) individuals aged between 20 and 60 years without medical contraindication to engage in physical exercise, (ii) possession of a smartphone (cell phone with Android operating system), and (iii) willingness to participate in the research by signing the informed consent form. The exclusion criteria were experience angina during exertion, history of acute myocardial infarction within the 12 months prior to the start of the protocol, require oxygen supplementation, exhibit clinical instability in the month preceding the protocol initiation, uncontrolled hypertension, visual diseases that prevent the performance of the protocol, chronic conditions hindering participation in the exercise protocol, illiterate, and refuse to sign informed consent form.

### Who will take informed consent? {26a}

A proficient researcher will administer the informed consent form, ensuring that participants comprehend and willingly agree to participate in the study.

### Additional consent provisions for collection and use of participant data and biological specimens {26b}

On the consent form, participants will be asked if they agree to use of their data should they choose to withdraw from the trial. Participants will also be asked for permission for the research team to share relevant data with people from the universities taking part in the research or from regulatory authorities, where relevant. This trial does not involve collecting biological specimens for storage.

## Interventions

### Explanation for the choice of comparators {6b}

The World Health Organization recommends engaging in physical activity as a preventive measure against various diseases and for promoting overall health. This recommendation will be implemented within the control group.

## Intervention description {11a}

### Phase 1: Functional assessment and biosensor validation

Following the development of the wearable biosensor designed for heart rate and step count monitoring, a validation test will be conducted during the incremental shuttle walk test (ISWT) [18, 19]. This ISWT is a recognized, reliable, and secure walking test for assessing functional capacity. Throughout the evaluation, participants will wear the biosensor on their nondominant wrist to capture HR and step data. For HR measurement, the Polar H10 chest strap (Polar Electro Oy, Kempele, Finland) will serve as the reference [20], while the triaxial Actigraph GT3X accelerometer (Actigraph, Pensacola, Florida) will be used on the nondominant wrist to measure step count [21].

The assessment will encompass several variables, including maximum distance, top speed, oxygen consumption (VO_2_), tissue oxygenation, and blood pressure (measured at the test’s outset and conclusion). Continuous HR tracking will be achieved by both the biosensor under evaluation and the Polar H10 monitor. Additionally, perceived exertion will be recorded using the modified Borg scale [22]; other data to be captured include the stage and corresponding stage at which the test is stopped. Two ISWTs will be conducted, with a maximum interval of one week between them. The results from the most successful trial will be utilized for subsequent analyses. This phase will be executed within a research laboratory environment by trained researchers.

#### Oxygen consumption

During ISWT, a comprehensive analysis of physiological responses will be conducted using the portable PNOĒ telemetry system (ENDO Medical, Palo Alto, CA). This system enables the assessment of variables like oxygen consumption (VO_2_) and carbon dioxide (VCO_2_) production through a breath-by-breath approach [23].

#### Muscle oxygenation

Employing the PortaMon spectrometer (Artinis Medical, Netherlands), alterations in oxyhemoglobin (O_2_Hb) and deoxyhemoglobin (HHb) chromophore concentrations will serve as indicators for assessing blood volume and oxygen consumption in the lower limb muscles. For this purpose, an optode will be affixed to the skin using adhesive tape and then covered with black tissue over the proximal third of the vastus lateralis muscle on the dominant limb. Throughout the ISWT protocol, the concentrations of O_2_Hb and HHb will be continuously monitored within the muscle [24, 25].

### Phase 2: Evaluation of the gamified application for prescription and physical training in a controlled environment

Phase 2 will entail a comprehensive examination of the gamified application’s effectiveness within a controlled laboratory setting. Assessments and interventions during this phase will be closely supervised by researchers. The functional test conducted during phase 1 will be replicated at both the outset and the conclusion of phase 2, with trained researchers overseeing the process.

#### Prescription of physical exercise

Building upon the ISWT results, the intensity of physical exercise will be tailored. The training protocol will span six weeks, with sessions conducted 5 days a week. The training volume will encompass a minimum of 300 min weekly at moderate intensity (40–60% of heart rate reserve) or a minimum of 150 min weekly at vigorous intensity (60–80% of heart rate reserve) [26]. The training will involve a one-step staircase (20 cm height, 60 cm length, and 40 cm width). The target intensity will range from 40 to 80% of the heart rate reserve, calculated based on ISWT. The heart rate reserve will be determined by subtracting the resting heart rate from the maximum heart rate achieved during the functional test. Throughout the exercise session, both HR monitors (Polar H10) and the test biosensor will be closely monitored.

The gamified smartphone application will offer visual feedback on intensity and heart rate, alongside researcher observation of heart rate measured by Polar H10 and the biosensor linked to the gamified application. Additionally, the biosensor, equipped with an accelerometer, will track step count during training. Phases within the scenario will be defined using gamification techniques, incorporating rewards, competitions, and other strategies to ensure adherence and engagement across varying intensities, including warm-up, maintenance, and cool-down phases.

In the initial week, sessions will last 30 min: a 5-min stationary gait warm-up, 20 min of step interval training (2 min at target HR and 3 min at low intensity), and a 5-min stationary gait cool-down. Over the subsequent 5 weeks, each session will extend to 60 min: a 5-min stationary gait warm-up, 50 min of step training at target HR, and a 5-min stationary gait cool-down. Sessions will occur at least twice a week and no more than five times. To meet the minimum adherence criterion, participants must achieve at least 70% of the prescribed training volume outlined in the protocol. Upon completing the 6-week training program, a new functional capacity assessment will be conducted.

#### Gamified application implementation

A mobile platform integrating gamification techniques will be deployed to ensure adherence to prescribed physical activity (PA) regimens, promoting user engagement. To select the most suitable platform, an analysis will be conducted of prevalent devices among the Brazilian population, as identified in PGB 2021 [27].

This system will enable the design of physical activities that ensure adherence, exercise intensity, and duration. Requirements will be categorized into functional and non-functional aspects. Functional requirements define the system’s functions and responses to specific inputs and behaviours, while non-functional requirements outline quality standards and limitations on system services.

The development tools chosen for this system are Blender 2.91 [28] for creating models, animations, and textures and Unity 2020 [29] for animation, direction, rendering, and programming. These tools were selected due to their extensive use in similar solutions.

Avatars will reflect individual physical characteristics, including factors like body fat levels, which directly impact the avatar’s appearance. Hair and facial hair customization will be offered with 15 distinct options for each. Additionally, the virtual store within the system will provide 15 clothing models for both genders.

Creating the scenarios involves compiling visual reference materials and adjusting scene elements to match common smartphone screen dimensions. Decoration pieces will be modelled for in-game scenarios and can be purchased using earned in-game coins obtained through exercising. An original soundtrack and synthesized sound effects will enhance the gaming experience, aligning with the game’s artistic direction.

All interactions between characters and scenario items will be programmed, encompassing actions like sleeping, eating, and exercising on various equipment like treadmills and bicycles. Engaging mini-games will be integrated into equipment-based exercises, offering players points redeemable for in-game store items. To engage in exercises, players will expend energy points earned through real-world physical activity.

### Phase 3: Evaluation of gamified in uncontrolled environment

Phase 3 comprises a noninferiority, investigator-blinded, randomized clinical trial with two distinct groups: the intervention and control groups. Participants within the intervention group (IG) will install the gamified application on their smartphones and use a biosensor during exercise sessions. These sessions will align with the prescribed intensity derived from the ISWT, following the 6-week regimen detailed in phase 2. Participants will be duly instructed to cease exercising if they experience symptoms like chest pain, discomfort, or dizziness.

Within the gamified application, participants will have access to a diary feature, enabling them to log notifications received and their efforts during exercise, both monitored by the biosensor and the gamified app. On the other hand, individuals in the control group (CG) will receive instructions to engage in physical activity at home without biosensor monitoring. They will use the app independently, aiming for a minimum of 300 min per week at moderate intensity or at least 150 min per week at vigorous intensity (self-assessed).

Preceding and following the 6-week period, both the IG and CG will undergo assessments akin to those conducted in phase 1. These include evaluations of functional capacity, muscle oxygenation, and further assessments led by trained and blinded researchers.

#### Endothelial function assessment

The evaluation of endothelial function will encompass two methods: pulse tonometry (EndoPAT2000, Itamar Medical, Caesarea Israel) [30–32] and ultrasonography to measure flow-mediated vasodilation (FMD) of the brachial artery (MyLab 70 Xvision, Esaote SpA, Florence, Italy) [33, 34].

#### Autonomic function assessment

The autonomic function of participants will be evaluated through the analysis of heart rate variability (HRV), involving the recording of R-R intervals utilizing the Polar H10 strap (Polar Electro Oy, Kempele, Finland) [21].

For HRV analysis, the time series of RR intervals, acquired from the Polar recordings, will undergo interpolation and decimation. This process aims to generate a time-equally spaced series, subsequently subjected to fast Fourier transformation (FFT) using a MATLAB-based algorithm (MATLAB 6.0, Mathworks Inc., USA). The spectral power will be calculated by integrating within each specified frequency band of interest. The RR time series will be explored in both time and frequency domains, including low frequency (LF), high frequency (HF), and very low frequency (VLF). This will yield parameters related to variability and autonomic balance.

In the time domain, computed parameters will encompass mean RR interval values, standard deviation, and the square root of the sum of the squares of successive differences (rMSSD).

### Criteria for discontinuing or modifying allocated interventions {11b}

Physical exercise may lead to effects such as increased heart rate, sweating, fatigue, minor discomfort related to equipment use, discomfort in the lower limbs, and mild muscular fatigue after treadmill training. In the event of any of these mentioned episodes occurring, whether during assessments or physical training, researchers will halt the protocol and provide initial assistance if necessary. If the participant stabilizes and agrees, the protocol may be resumed.

During the execution of training in an uncontrolled environment (phase 3), participants will be advised to discontinue the protocol if any discomfort or symptoms arise. They should await the cessation of these symptoms. If conditions permit, participants should resume from where they left off. If there is no improvement, they will be instructed to contact the researchers via telephone for appropriate measures.

### Strategies to improve adherence to interventions {11c}

Following participant randomization, the research team will refrain from implementing specific operational measures to ensure the desired follow-up level. This approach aligns with one of the study’s primary objectives, which is to assess exercise adherence facilitated by gamification. What will be done is to send a standard message weekly to both groups requesting weekly recording of the activity carried out during the week in a spreadsheet.

### Relevant concomitant care permitted or prohibited during the trial {11d}

Participants in the study will be advised against participating in any other exercise programs simultaneously.

### Provisions for post-trial care {30}

The investigators will bear the responsibility of providing assistance to participants who experience any harm during the execution of the protocol.

### Outcomes {12}

Primary outcomes will encompass the following: (1) the concordance between HR and step count measurements assessed by the biosensor under test compared to standard devices (Polar H10 and ActiGraph, respectively); (2) participant perception regarding the utility of the gamified application for exercise engagement; (3) comparison with the prevalence of adherence to the physical activity protocol between the control and intervention groups; (4) evaluation of exercise capacity through ISWT, including stage and the corresponding phase at which the test was halted; and (5) measurement of oxygen consumption (VO_2_).

Secondary outcomes will include the following: (1) tissue oxygenation assessed via near-infrared spectroscopy (NIRS), (2) subjective perception of exertion during ISWT, (3) evaluation of endothelial function, and (4) assessment of autonomic function.

### Participant timeline {13}

**Fig. 2:**
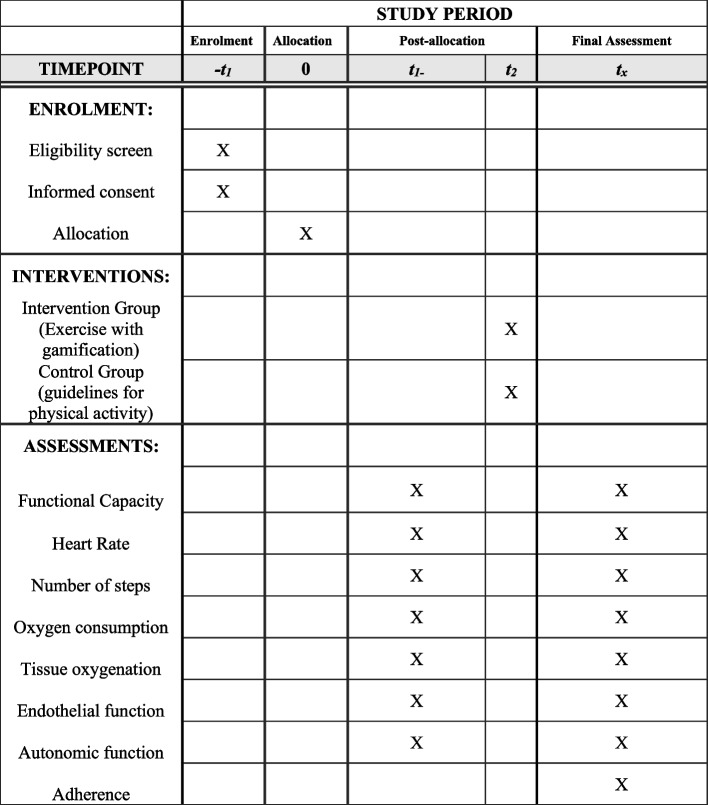
Schedule of enrolment, interventions, and assessments

After randomization, participants will perform the initial assessment and in the following 6 weeks will perform the exercise prescribed according to the allocation group. After 6 weeks, the assessment will be repeated and participation in the study will end. The chronological schedule for participants can be found in Fig. 2.

### Sample size {14}

Since phases 1 and 2 consist of a pilot study for the clinical evaluation of a biosensor prototype and the gamified application that considers a biofeedback system for performing physical exercises, a convenience sample of 15 individuals without medical contraindications for PA will be studied. Assuming a sample loss of 30%, we will recruit 20 participants.

The third phase will be a noninferiority, investigator-blinded randomized clinical trial of the individuals eligible for the study—considering the same eligibility criteria as in phases 1 and 2, in addition to being sedentary—will be divided into two groups: the intervention group (IG), which will receive the exercise protocol through the biosensor and gamified application, and the control group (CG), which will only receive guidelines for performing PA. Considering a confidence level of 95%, power of 80%, ratio of 1:1 between the CG and IG, and estimated prevalence of adherence to the protocol of 70% and 50% for the IG and CG, respectively, it would be necessary to allocate 104 individuals in each group. Adding 30% for possible follow-up losses, 149 sedentary individuals will be assigned to each group, totalling 298 subjects in the study.

### Recruitment {15}

The recruitment of sedentary individuals will involve a cross-sectional investigation that will utilize the International Physical Activity Questionnaire (IPAQ), which assesses the frequency, duration, and type of physical activity an individual engages in across various settings, such as work, transportation, leisure time, and domestic chores. This outreach endeavour will be geared towards the broader population, focusing on identifying potential eligible candidates for the study within a specific city in southern Brazil. To achieve random allocation between the CG and the IG, a preliminary cross-sectional study targeting the general population will be executed. This study aims to pinpoint sedentary individuals suitable for inclusion in the randomized clinical trial.

Consequently, considering a confidence level of 95%, a power of 80%, an estimated sedentary lifestyle prevalence of 50%, and a margin of error of 2 percentage points, it will be imperative to interview 2875 individuals (factoring in a 20% allowance for possible losses and refusals). This approach is designed to successfully identify an adequate number of sedentary individuals for participation in the randomized trial’s phase 3.

## Assignment of interventions: allocation

### Sequence generation {16a}

We will employ a computerized random number generator to allocate individuals to either the IG or the CG in a 1:1 ratio. Given the potential variability in VO2 values based on age and gender, we have chosen to utilize block randomization. This method will help us ensure that there is a comparable distribution of age and gender between the two groups, thereby mitigating any potential bias.

### Concealment mechanism {16b}

The allocation sequence for this process will be implemented using sealed envelopes to maintain the integrity of the randomization process.

### Implementation {16c}

Notably, the researcher entrusted with generating the allocation sequence will remain uninvolved in participants’ data collection. Additionally, the investigator responsible for conducting the functional assessments will be kept blind to the participants’ allocation status.

## Assignment of interventions: blinding

### Who will be blinded {17a}

Owing to the nature of our intervention, which encompasses physical activity and gamification, participant blinding will not be feasible. However, the researcher overseeing the evaluation process will be kept blinded.

### Procedure for unblinding if needed {17b}

Unblinding will not be permitted at any point during the study due to its inherent design and the nature of the interventions involved.

## Data collection and management

### Plans for assessment and collection of outcomes {18a}

Participants will be provided transportation vouchers for their visits to Universidade Federal de Ciências da Saúde de Porto Alegre. Reminder calls will be placed to inform participants of their assessment days. During the intervention, no proactive contacts will be initiated to enhance adherence, as adherence is one of the evaluated outcomes.

### Plans to promote participant retention and complete follow-up {18b}

Since one of the study’s goals is to assess the impact of gamification on adherence to PA, no specific measures will be undertaken during the study to bolster adherence. Upon the study’s conclusion, all participants will be contacted for a final assessment. For measuring adherence, the intervention group will register their adherence via a dedicated application. Meanwhile, the control group will be guided to record their physical activities on a weekly basis added to plans to promote participant retention and complete follow-up.

### Data management {19}

Characterization data collection and evaluation result recording will be documented on paper forms. Subsequently, the principal investigator or their delegate will input the data into an Excel spreadsheet. Another investigator will review this data entry to mitigate potential errors.

### Confidentiality {27}

All participant information will be securely stored in locked file cabinets, located in restricted-access areas, for a duration of 5 years post-study completion.

### Plans for collection, laboratory evaluation, and storage of biological specimens for genetic or molecular analysis in this trial/future use {33}

See the “ Additional consent provisions for collection and use of participant data and biological specimens {26b}” section; there will be no biological specimens collected.

## Statistical methods

### Statistical methods for primary and secondary outcomes {20a}

Data distribution will be assessed using the Kolmogorov–Smirnov test. Parametric data will be presented as mean ± standard deviation, while non-parametric data will be displayed as median (interquartile range). In phase 1, resting and maximum HR assessed by both the Polar H10 monitor and the test biosensor will be compared using either *t*-test or Wilcoxon test, based on data distribution. The same comparison will be applied to the step count measured by the test biosensor and the ActiGraph triaxial accelerometer. Intraclass correlation coefficient (ICC) will also be computed, along with Bland–Altman analysis to assess HR and step count agreement.

During phase 2, a quantitative analysis will be conducted on the frequency of possible gamified application failures. Pre- and post-exercise protocol data, including functional capacity, VO_2_, tissue oxygenation, maximum and resting HR, systemic blood pressure, and perceived exertion, will be compared using *t*-test or Wilcoxon test, based on data distribution. Similarly, the number of steps evaluated by the biosensor under test and the ActiGraph accelerometer will undergo the same analysis, along with ICC calculation.

For phase 3, a qualitative analysis will be performed on participants’ diary reports and a quantitative assessment of potential gamified application failures. Step count measured by the biosensor will be compared to the ActiGraph accelerometer using Student’s *t*-test or Wilcoxon rank-sum test, depending on residual distribution. Agreement between step counts will be evaluated using Bland–Altman test, Lin’s correlation coefficient, and intraclass correlation coefficient. Pre- and post-exercise protocol data, encompassing functional capacity, VO_2_, tissue oxygenation, maximum and resting HR, systemic blood pressure, subjective exertion perception, endothelial function, and HRV, will be analysed using Student’s *t*-test for paired samples or Wilcoxon signed-rank test, aligned with residual distribution. Participants achieving a minimum of 70% completion of the prescribed protocol will be classified as adherent. As the random allocation is anticipated to ensure baseline comparability between the control and intervention groups, unadjusted analyses will primarily be carried out. In the event of any non-comparability in baseline characteristics due to chance, appropriate adjustments will be made to prevent potential biases. Significance level will be set at *p* < 0.05. The data will be analysed using Statistical Package for the Social Sciences (SPSS) 20.0 (SPSS, Chicago, IL, USA).

For the progression of our study through its phases, we have set clear criteria to determine the acceptability of outcomes: *Phase 1*: We regard the study as satisfactory if the standard error between the biosensor under test and the reference measurements is within 5%. If this criterion is not met, we would reconsider moving to the next phase until the discrepancies are addressed. *Phase 2*: The study’s progression will depend on the functionality of the application. Should there be serious errors within the application that prevent exercise or significantly hinder adherence, we would need to address these issues before considering progression to phase 3. *Phase 3*: Here, our benchmark for continuation is achieving at least 70% adherence. Any value below this would prompt us to assess the reasons for the reduced adherence and potentially re-evaluate our approach. Additionally, it is important to note that throughout all phases, we have ensured that there are no operational issues related to the app. Thus, significant data recording failures in earlier phases would indeed influence our decision to proceed to the next phase.

### Interim analyses {21b}

No interim analyses are planned throughout the duration of the study.

### Methods for additional analyses (e.g. subgroup analyses) {20b}

No additional analyses are planned.

### Methods in analysis to handle protocol non-adherence and any statistical methods to handle missing data {20c}

Intention-to-treat (ITT) analyses will be carried out for the primary outcomes to uphold the initial randomization and maintain the real-world context of participants’ treatment assignments. This approach involves including all randomized participants within their originally allocated groups, ensuring the preservation of the study’s initial design.

### Plans to give access to the full protocol, participant-level data, and statistical code {31c}

If deemed necessary, the corresponding author will provide access to the complete protocol, participant-level dataset, and statistical code.

## Oversight and monitoring

### Composition of the coordinating centre and trial steering committee {5d}

The biosensor prototype development involves Universidade do Vale do Rio dos Sinos and Universidade Federal do Rio Grande do Sul, while the gamified application is a task for Universidade Federal do Pará. Universidade Federal de Ciências da Saúde de Porto Alegre will handle data collection and analysis. The roles and responsibilities are as follows: the coordinating centre (Universidade Federal de Ciências da Saúde de Porto Alegre) is responsible for overseeing the overall progress of the trial, ensuring adherence to protocols, and liaising with involved universities. The trial steering committee will guide the direction of the trial, ensure milestones are met, and review progress. Day-to-day support is as follows: Universidade Federal de Ciências da Saúde de Porto Alegre will be the primary institution providing daily operational and organizational support for the trial. Meeting frequency is as follows: the group responsible for daily operations meets by-monthly to review progress, address concerns, and plan for upcoming tasks.

### Composition of the data monitoring committee, its role and reporting structure {21a}

Given the trial’s single-centre nature, we have not instituted a separate data monitoring committee. All data collection procedures will occur at Universidade Federal de Ciências da Saúde de Porto Alegre and be supervised by the research coordinator (PD).

### Adverse event reporting and harms {22}

Any adverse events that occur after enrolment in the study will be documented, and the research team will provide assistance to participants who experience harm during the protocol implementation.

### Frequency and plans for auditing trial conduct {23}

We did not consider a trial steering group and independent data monitoring and ethics committee given the low-risk nature of the intervention. The project management group will meet monthly to review trial conduct. The main researcher (PD) will be responsible for monthly monitoring of research conduction and ethical adequacy.

### Plans for communicating important protocol amendments to relevant parties (e.g. trial participants, ethical committees) {25}

Any adjustments to the protocol that could influence the execution of the investigation, encompassing alterations in research goals, research structure, or methodologies, will necessitate an official revision to the protocol.

### Dissemination plans {31a}

The outcomes of the research will be showcased in both domestic and international scientific gatherings, in addition to being featured in peer-reviewed journals on a national and global scale. The attribution of authorship in any publications will adhere to the four criteria outlined by the International Committee of Medical Journal Editors.

## Discussion

This study focuses on evaluating the development and integration of innovative and safe technologies, such as biosensors and apps, to ensure appropriate intensity during prescribed physical exercises. The aim is to enhance individuals’ adherence to physical activity, monitor health biomarker responses (heart rate and steps), and to examine the effects of combining gamification with wearable activity devices to improve exercise engagement.

### Gamification’s role in promoting PA

Gamification interventions have shown potential in boosting PA participation and self-monitoring while also promoting a sense of enjoyment. However, the literature lacks exploration of the impact of combining gamification with wearable activity devices for promoting PA, as evidenced by divergent findings from a recent systematic review [16]. This study aims to address this gap by investigating the effects of this combination. The hypothesis is that merging gamification with wearable devices can lead to improved adherence to exercise routines.

### Reviving enjoyment through gamification

The concept of “serious games” has evolved to a point where the element of fun has diminished, affecting adherence [35]. In response, our study’s gamification approach seeks to reintroduce the enjoyment associated with physical exercise, making the activity pleasurable and facilitating adherence. Key aspects of our gamification strategy involve incorporating game elements, utilizing feedback, and setting goals to enhance the effectiveness and perceived reliability of the approach [35].

### Gamification’s evolving role in health care

Since 2010, gamification has garnered interest among researchers as a tool to enhance engagement in healthcare. However, the existing literature lacks sufficient publications to definitively establish the efficacy of gamification in e-Health contexts [36]. If successful, the outcomes of this trial will furnish the necessary evidence to inform future public health decisions related to exercise practices. The expectation is that the app will foster engagement and bolster adherence to PA, addressing a significant challenge. Moreover, the integration of biosensors into the gamification framework will elevate exercise intensity, accuracy, and safety.

In conclusion, this study endeavours to bridge gaps in knowledge by examining the effects of combining gamification with wearable devices in the context of exercise promotion. The anticipated outcomes include improved exercise adherence, engagement, and intensity, thereby contributing valuable insights for shaping future approaches to public health interventions aimed at enhancing physical activity levels.

### Limitations of the study

The study acknowledges several inherent limitations. Primarily, our choice to encompass a broad age range presented challenges, particularly in app communication. Research consistently indicates that distinct age groups, from the elderly to younger individuals, have varying preferences in terms of language use, visual aesthetics, and technological interface design. Elderly participants might find modern app interfaces or overwhelming, while younger participants could desire more dynamic and interactive features. Additionally, individuals in the aged bracket occasionally face challenges in adapting to rapidly evolving technological platforms, which might lead to frustration. This could deter them from consistently engaging with a gamified exercise application. While this age diversity was intentional to achieve a comprehensive understanding, it inadvertently introduced complexities in app design experience. Future iterations of the study might benefit from tailored app versions or additional training sessions to bridge this technological gap across age groups.

## Trial status

The recruitment phase for the exploratory study was initiated in August 2022. Looking ahead, the recruitment process for the randomized clinical trial is scheduled to commence in June 2023 and is anticipated to conclude around December 2026. Although the protocol submission took place, a protocol number has not yet been assigned. This ongoing effort aims to rigorously investigate the outlined objectives, contributing valuable insights to the field.

## Data Availability

Data can be provided upon a reasonable request.

## Abbreviations

CG: Control group
FFT: Fast fourier transformation
FMD: Flow-mediated vasodilation
HHb: Deoxyhaemoglobin
HR: Heart rate
HRV: Heart rate variability
ICC: Intraclass correlation coefficient
IG: Intervention group
IoT: Internet of things
ISWT: Incremental shuttle walk test
ITT: Intention-to-treat
LPWH: Low power wide area network
O_2_H_b_: Concentration of oxyhaemoglobin
PA: Physical activity
REBEC: The brazilian registry of clinical trials
SPIRIT: Standard protocol items recommendations for interventional trials
SPSS: Statistical package for the social sciences
VCO_2_: Carbon dioxide
VO_2_: Oxygen consumption
WHO: World health organization
